# Fracture Resistance of Roots With Si̇mulated Internal Root Resorption Followi̇ng Repair With Calcium Silicate‐Based Cements

**DOI:** 10.1155/ijod/2877033

**Published:** 2026-02-08

**Authors:** Havva Gozde Sen, Ayca Yilmaz

**Affiliations:** ^1^ Department of Endodontics, Faculty of Dentistry, Istanbul Beykent University, Istanbul, Türkiye, beykent.edu.tr; ^2^ Department of Endodontics, Faculty of Dentistry, Istanbul University, Istanbul, Türkiye, istanbul.edu.tr

**Keywords:** Biodentine, BioMTA+, NeoMTA 2, ProRoot MTA, root resorption

## Abstract

This study aimed to evaluate the fracture resistance of teeth with simulated internal root resorption cavities filled with calcium silicate‐based cements (CSCs). Seventy‐two extracted central incisor teeth had their crowns removed to standardize the root length to 14 mm. The teeth were randomized into six groups (*n* = 12 each), including a positive control (with no simulated resorption cavities) and negative control (with no filling) group. To simulate internal root resorption cavities, cavities were made along the 6 mm section using a number 10 round bur on groups. In four groups, artificially created resorption areas were filled with ProRoot MTA, Biodentine, NeoMTA 2, and BioMTA+. The specimens were subjected to fracture resistance testing using a universal testing machine (UTC). Compressive 135° angle loading was applied to the roots at a rate of 1 mm/min. The fracture resistance was recorded in Newtons and statistically analyzed using a one‐way analysis of variance (Welch’s test) and Tamhane’s T2 test. The fracture resistance was significantly higher in the Biodentine group than in the negative control group. Therefore, the use of Biodentine in internal root resorption cavities may, to some extent, contribute to preserving or supporting the tooth structure.

## 1. Introduction

Dentin thickness is a critical factor in determining fracture resistance. Dentin loss reduces the fracture resistance of teeth to occlusal or traumatic forces [1]. Therefore, root‐filling materials and calcium silicate‐based cement (CSC) may reinforce internal root resorption cavities [2]. Root‐filling materials should also focus on reinforcing the mechanical strength of the tooth; otherwise, the tooth may remain highly susceptible to fracture despite an apparently successful endodontic outcome [2].

Root resorption refers to the excessive loss of dentin and cementum due to the stimulus‐mediated activation of osteoclastic cells, and the ongoing activity of these osteoclastic cells plays a crucial role in this process. Regardless of the initial cause, resorptive processive destruction is generally inflammatory [3]. Internal root resorption typically initiates within the root canal and can destroy intraradicular dentin, affecting the integrity of the root canal structure. Internal root resorption can manifest anywhere along the root canal and often presents radiographically as an oval‐shaped enlargement within the pulp. Detecting internal root resorption may be challenging until the root resorption cavities have significantly advanced, resulting in perforation or symptoms of apical periodontitis after the pulp has undergone necrosis [3, 4]. Although internal root resorption is rare, its main causes are trauma, periodontal infections, caries‐related pulpitis, predentin damage caused by excessive heat during restorative procedures in teeth with vital pulp, root resections of teeth with healthy pulp, autotransplantation, orthodontic treatment, broken teeth, calcium hydroxide pulpotomy, pulp amputation, and idiopathic dystrophic changes in teeth with vital pulp [4]. Internal root resorption is asymptomatic and can be detected by routine radiography. In the literature, cone‐beam computed tomography (CBCT) has been reported to be a reliable and definitive method for the identification of internal root resorption and treatment planning. The presence, location, and size of perforation of the internal root resorption cavities can be seen on CBCT scans [4, 5]. If the root weakens with the risk of perforation, the root canal is filled with gutta‐percha cones and resorption cavities with CSCs [2].

CSCs were first introduced in the field of dentistry in 1993. CSCs are hydraulic cements composed primarily of tricalcium silicate, dicalcium silicate, and tricalcium aluminate. Calcium silicate‐based materials strengthen teeth primarily through their tricalcium silicate (Ca_3_SiO_5_) content, which releases high levels of calcium ions during hydration. These ions promote apatite formation and the development of mineral “tag‐like structures” that create a micromechanical and chemical bond with dentin, increasing its microhardness and fracture resistance. The accompanying formation of calcium hydroxide and C–S–H gel further supports mineral deposition and structural reinforcement [6]. In this study, we used the following CSCs: ProRoot MTA, Biodentine, NeoMTA 2, and BioMTA+. Mineral trioxide aggregate is used as CSC owing to its suitable physical properties and setting time. It stimulates dentin development because of its low cytotoxicity and good biocompatibility [7]. ProRoot MTA is a CSC consisting of powder–liquid, which has a high pH but disadvantages such as long setting time and tooth discoloration [7]. BioMTA+ is a newly developed calcium silicate‐based material engineered to enhance dentin reinforcement and bioactive performance. The manufacturer claims that BioMTA+ has high biocompatibility with the hydroxyapatite content. This CSC was developed by Cerkamed [8]. NeoMTA 2 is a new CSC, consisting of powder and liquid, and has been used recently owing to its fast setting time and ability to repair periapical tissue [9]. To overcome the disadvantages of MTA, a new CSC, Biodentine (Septogon, Saint‐Maur‐des‐Fossés, France), was introduced in 2009. The manufacturer claims that this biomaterial has properties similar to those of MTA. Arguably, their physical and chemical properties are superior [10]. It has been suggested that Biodentine has better bonding abilities through forming an interfacial layer [2, 11].

The fracture of teeth that have undergone endodontic treatment often results from the progression of microcracks, which have a tendency to propagate [12]. The success of endodontic treatment can vary based on the stresses encountered within the oral cavity, including those arising from parafunctional and functional habits of the teeth [13]. To assess fracture strength, a universal testing machine (UTC) is employed for conducting compressive strength fracture tests. This machine systematically applies a predetermined force along a specified axis to the test materials, records the final fracture force in Newtons (N) and transfers these data to the software for analysis. Various tip shapes can be utilized within the testing device, ensuring that compressive loads are uniformly distributed when assessing the fracture resistance of teeth [14, 15]. The null hypothesis for the present study posits that the new CSCs, specifically NeoMTA 2 and BioMTA+, exhibit fracture resistance values similar to those of the other CSCs examined in this study.

## 2. Methods

This study was conducted at Istanbul University, Faculty of Dentistry, Department of Endodontics. This work was supported by the Scientific Research Projects Coordination Unit of the Istanbul University. Ethical approval for this study was obtained from the Istanbul University Scientific Research Ethics Committee (Decision Number: 28678:2020). A total of 72 central maxillary incisors were extracted. A power analysis determined a sample size of 12 teeth per group. All teeth were single‐rooted with a single canal and were checked radiographically. The tissues on the periodontal surfaces of the teeth were cleaned and stored in distilled water at room temperature. With the aim of standardization, one observer, using a microscope (Leica MZ7.5, Bensheim, Germany) under 20× magnification, selected teeth with no cracks or fracture lines. At the cementoenamel junction, the mesiodistal and buccolingual diameters of the teeth were measured using a digital caliper, and teeth exhibiting more than a 20% difference between these dimensions were excluded from the study.

To achieve a standardized root length of 14 mm, all the teeth underwent decoronation. Subsequently, the working length of the 72 roots was determined to be 1 mm shorter using a #10 K‐file (Mani Inc., Tochigi‐Ken, Japan). The removal of pulp tissue was carried out with #15 and #20 K‐files. Root canal preparation involved the use of Reciproc R40 instruments (WDV, Munich, Germany). Throughout the shaping process, the root canals were irrigated with 2 mL of 5.25% sodium hypochlorite (NaOCl) (Cerkamed, Stalowa Wola, Poland) following the use of each canal instrument, employing a 30‐gauge needle for irrigation. After the completion of instrumentation, each root was irrigated with 5 mL of 5.25% NaOCl and 5 mL of 17% EDTA (Cerkamed, Poland) to remove the smear layer, followed by rinsing with 5 mL of distilled water. Subsequently, the root canals were dried using an R40 paper point (WDV, Munich, Germany).

The positive control group consisted of 12 roots that did not exhibit any internal resorption areas. In addition, the positive control group had their root canals filled with Reciproc R40 gutta‐percha cones (WDV, Munich, Germany) and AH Plus sealer (Dentsply, Konstanz, Germany).

To simulate internal root resorption cavities, cavities were made along the 6 mm section using a number 10 round bur on a total of 60 roots. After cavity preparation, each root was examined under × 10 magnification using a dental operating microscope to confirm that the cavity length and position corresponded to the predetermined 6‐mm segment. Any specimens in which the cavity dimensions deviated from the standardized size were excluded and replaced to maintain uniformity across the sample. Following this, the apical 8 mm of these 60 roots was filled with Reciproc R40 gutta‐percha cones along with AH Plus sealer. Subsequently, these 60 roots were divided into five groups, each containing 12 roots (*n* = 12), as detailed below. To prepare the roots for the filling procedure, any excess gutta‐percha cones protruding from the starting point of the resorption area were removed using the Fast Pack (Eighteeth, Changzhou, China). Ultimately, 48 simulated internal resorption cavities were filled with different types of CSCs.

Group 1: The apical 8 mm of the root canals was filled with Reciproc R40 gutta‐percha cones and AH Plus sealer. The resorption cavities were filled with Biodentine (lot number B26176). Biodentine is a capsule containing powder. A single capsule containing 0.7 g powder was mixed with five drops of calcium chloride‐containing liquid to serve as an accelerator for 30 s in a mixing device (Amalgamator; Fushion SyG‐200, TRIUP International Corporation, Shanghai, China) at 4000 rpm. Internal resorption cavities were examined radiographically (Figure 1A).

Figure 1(A) Representative radiographic image of group Biodentine + gutta‐percha cones. (B) Specimens embedded in acrylic blocks.

**Figure:**
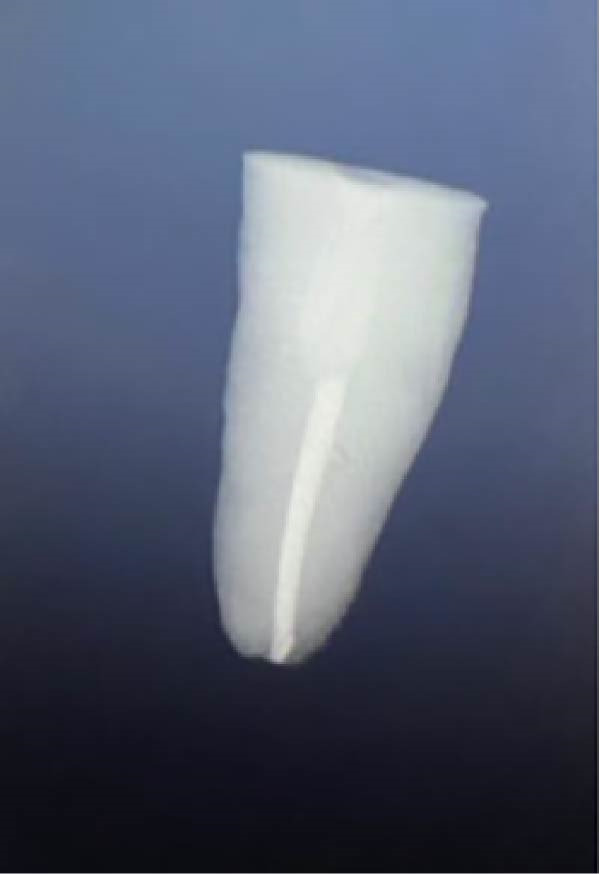
(A)

**Figure:**
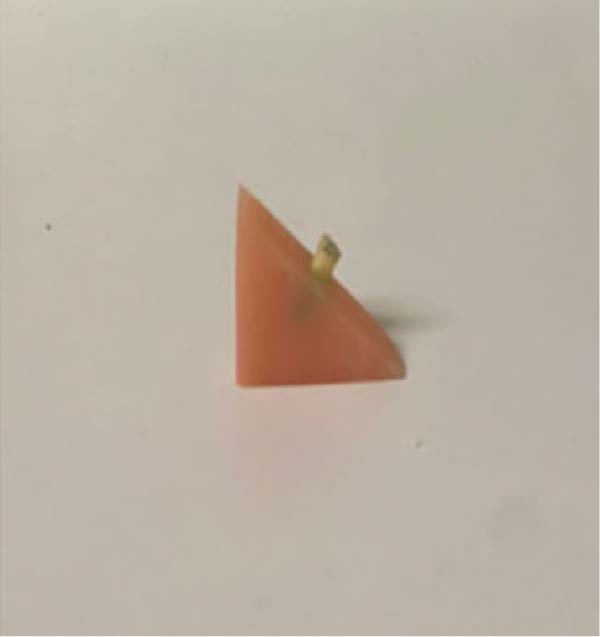
(B)

Group 2: The apical 8 mm of the root canals was filled with R40 gutta‐percha cones and AH Plus sealer. The resorption cavities were filled with ProRoot MTA (lot number 0000249679) using a hand plugger. ProRoot MTA was mixed with 0.5 g powder and a micro‐dose ampoule of liquid. The internal resorption cavities were examined radiographically.

Group 3: The apical 8 mm of the root canals was filled with Reciproc R40 gutta‐percha cones and AH Plus sealer. The resorption cavities were filled with NeoMTA 2 (lot number 2021031603) using a hand plugger. NeoMTA 2 was mixed in a 1 (0.1 g):1 powder/liquid ratio according to the manufacturer’s instructions. The internal resorption cavities were examined radiographically.

Group 4: The apical 8 mm of the root canals was filled with Reciproc R40 gutta‐percha cones and AH Plus sealer. The resorption cavities were filled with BioMTA+ (lot number 1808211) using a hand plugger. BioMTA+ was mixed in a 1 (0.14 g):1 powder/liquid ratio for 30 s according to the manufacturer’s instructions. The internal resorption cavities were examined radiographically.

Group 5 (positive control group): Teeth without resorption cavity when the root canals were instrumented with Reciproc R40. Root canals were filled with Reciproc R40 gutta‐percha cones and AH Plus sealer.

Group 6 (negative control group): The apical 8 mm of the root canals was sealed using Reciproc R40 gutta‐percha cones and AH Plus sealer. However, resorption cavities were deliberately created but intentionally left unfilled with any CSCs.

Subsequently, all roots were placed in Eppendorf tubes and were stored in a 100% humidity environment at 37°C for 7 days to allow for the complete setting of the cements. In our study, the apical 7 mm of all the roots was covered with polyvinylsiloxane‐based type A silicone (Elite HD+, Zhermack SpA, Rovigo, Italy) to simulate the periodontal ligament.

**Figure 2:**
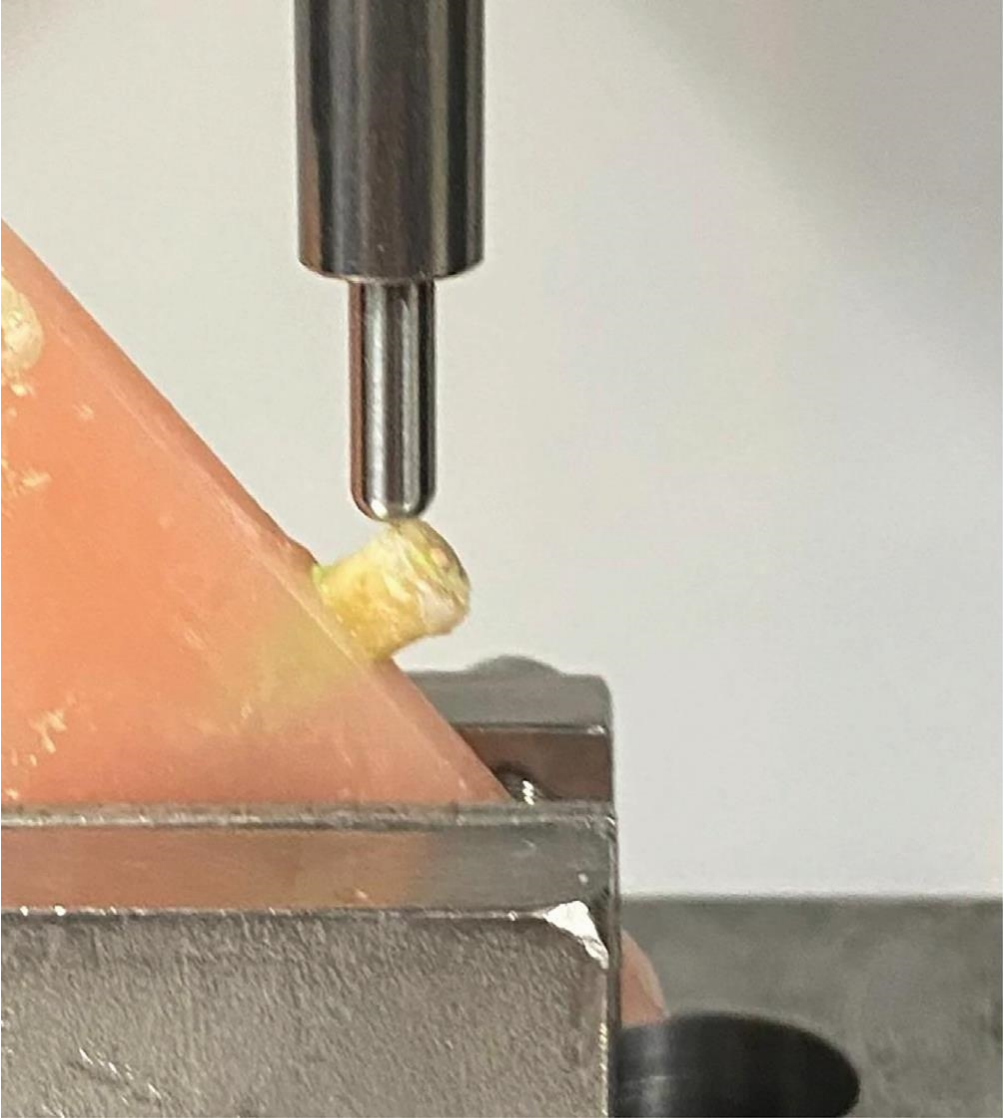
Specimens placed in a universal testing machine.

The roots were placed on right triangular acrylic resin blocks (Imicryl, Konya, Turkey) (Figure 1B). The apical 7 mm of all the roots was vertically mounted within these acrylic blocks. This mounting was carried out after utilizing a UTC (Autograph AG‐X; Shimadzu Corporation, Kyoto, Japan) (Figure 2).

The acrylic blocks were positioned within a UTC, and a 5 mm stainless spherical steel tip was brought into contact at a 135° angle to the long axis of the tooth, with a crosshead speed of 1 mm/min until fracture. Fracture values were recorded in newtons. Data were analyzed using IBM SPSS V23, utilizing a one‐way analysis of variance (Welch’s test) and Tamhane’s T2 test, with a significance level set at 0.05.

The fracture pattern was divided into three types. First, root fractures occurring above the acrylic surface were categorized as supracrestal root fractures. Root fractures below the acrylic level were classified as subcrestal root fractures [2]. Finally, if a fracture occurred along the long axis of the root, it was defined as a vertical root fracture. Types of fracture were analyzed utilizing Pearson Kikare test, with a significance level set at 0.05.

## 3. Result

The means, standard deviations, and minimum and maximum values of the fracture strength tests are presented in Table 1. Notably, the significant disparity was observed between the negative control group and the Biodentine group, with the Biodentine group exhibiting a statistically higher mean fracture value than the negative control group. In contrast, no significant variations were observed in the mean values among the other groups. It is worth noting that, while the differences were not statistically significant, the Biodentine group demonstrated the highest fracture strength among all groups. However, it is important to note that both NeoMTA and BioMTA+ did not exhibit fracture resistance values significantly different from the other CSCs, and their performance was comparable. The majority of the fracture patterns were situated above the level of the acrylic resin base, indicating supracrestal root fractures. There is no statistically significant difference between groups. The fracture patterns of the samples are listed in Table 2.

**Table 1:** Mean fracture resistance values (Newtons) and standard deviations for experimental groups (*n* = 12).

Groups	Fracture resistance (*N*) (Mean ± SD	Minimum–maximum (*N*)	*p*
Negative control	727.53 ± 227.63^a^	(316.41–1070.94)	—
Positive control	912.67 ± 227.8^ab^	(622.34–1269.06)	—
ProRoot MTA	1016.73 ± 236.24^ab^	(546.56–1344.06)	0.014
Biodentine	1075.21 ± 227.43^b^	(624.06–1402.19)	—
BioMTA+	861.03 ± 89.04^ab^	(744.84–981.88)	—
NeoMTA 2	897.38 ± 129.38^ab^	(676.25–1122.34)	—

*Note:* One‐way analysis of variance (Welch’s test) and Tamhane’s T2 test.

Different superscript small letters represent significant difference (*p*  < 0.05).

**Table 2:** Types of fractures.

Groups	Supracrestal fracture	Subcrestal fracture	Vertical fracture	*p*
Negative control	9	2	1	—
Positive control	5	5	2	—
ProRoot MTA	6	4	2	0.894
Biodentine	9	2	1	—
BioMTA+	8	3	1	—
NeoMTA 2	8	3	1	—

*Note:* Pearson’s chi‐square test. Different superscript small letters represent significant difference (*p*  < 0.05).

## 4. Discussion

This study aimed to compare fracture resistance with simulated internal root resorption cavities and to define fracture patterns. The null hypothesis of the present study was accepted because NeoMTA 2 and BioMTA+ showed similar fracture resistance values to other CSCs. The irrigation protocol, root canal shaping, and root canal filling methods can change the fracture resistance of the specimens [16, 17]. Therefore, all root procedures were performed by a single investigator following a standardized protocol.

In many studies, the roots were used for fracture resistance comparison [1, 2, 16]. First, fracture resistance was compared between the root and crown. Second, the teeth were decoronated, removing the crown, to prevent cervical defects from affecting the fracture resistance of the teeth [2, 18, 19]. In this study, the crowns were decoronated to a root length of 14 mm like other studies.

Several methods are available for creating simulated internal root resorption cavities. In a previous study, cavities were created to simulate resorption cavities by proceeding in a straight direction from the canal using round burs up to half of the root length [2]. Similarly, our study simulated root resorption areas. Internal root resorption is most common in the upper central incisors [3]. Based on this information, the upper central incisors were selected to simulate the internal root resorption cavities in this study.

In the previous study, it was suggested that CSCs are chemically bonded to the root dentin [11, 20]. Although the positive association between bond strength and fracture resistance is not clear, it is generally accepted that successful adhesion of cement to the root dentin increases its reinforcing effect [11, 19]. Therefore, MTA is an ideal repair material for teeth with internal root resorption. MTA possesses several advantageous properties, including good sealing capabilities, bactericidal properties, and ideal radiopacity. Although MTA is ideal, MTA types that can be used instead of materials have been the subject of discussion [21].

Upon analyzing the findings of our study, it was evident that there were no significant differences in fracture resistance among Biodentine, ProRoot MTA, NeoMTA 2, and BioMTA+. However, Biodentine showed significantly higher fracture resistance when compared to the negative control group. The differences in the size of the particles in the CSCs, the agents used as radiopacifiers and the powder/liquid ratios may result in differences in the fracture resistance in the compressive strength test [11, 22]. In the present study, although Biodentine reinforced the roots with simulated internal resorption cavities compared with the MTA groups, no significant difference was observed [23, 24]. In our study, Biodentine had a fracture resistance of 1075.21 ± 227.4 N. Various fracture resistance values ranging between 529.02 N and 1027.3 N were reported for Biodentine [1, 23]. In our study, while Biodentine‐reinforced simulated internal resorption cavities were better than MTA, the difference was not statistically significant. The higher fracture resistance of Biodentine in short‐term fracture resistance comparison studies is due to the similarity of Biodentine flexion strength to dentin [23]. In this study, Biodentine showed a higher fracture resistance than ProRoot MTA, with ProRoot MTA presenting a fracture resistance value of 1016.73 ± 236.24. This aligns with results from other studies [23, 25]. The particle size of ProRoot MTA is 2.44–3.05 µm, while the dentin tubule diameter is between 0.9 and 2.5 µm. This difference prevents ProRoot MTA from penetrating the dentinal tubules [11]. However, Biodentine penetrates the dentin structure, forming an interfacial layer immediately under the Biodentine cement. This layer is known as the mineral infiltration zone. This adhesion to the Biodentine dentin tubules may be one of the reasons for the high fracture resistance observed in our study [26].

Currently, BioMTA+ is used in dentistry. The manufacturers stated that these materials contained small‐grained hydroxyapatite. It has been claimed that this material will exhibit more durability than traditional MTA [27]. In this study, no significant differences were observed in CSCs fracture resistance.

BioMTA+ exhibited a fracture resistance value of 861.03 ± 89.04 in this study. BioMTA+ contains bismuth oxide and zirconium oxide as the radiopacifiers. Biodentine contains only zirconium oxide as the radiopacifier. Zirconium oxide has been reported to increase the fracture strength of material [21]. Another study claimed that bismuth oxide‐containing CSC increased the porosity of Portland cement, thereby reducing fracture resistance [28]. In this study, although BioMTA+ showed no significant differences, it showed a lower fracture strength than Biodentine. This may be due to the addition of bismuth oxide to BioMTA+. In addition, the higher fracture resistance of Biodentine than that of ProRoot MTA may be due to the zirconium oxide content of Biodentine and the bismuth oxide content of ProRoot MTA.

The direction of the applied forces varied in the other study [24, 29]. Predominantly, the fracture pattern in the roots was situated in the coronal portion due to thin dentin walls in the resorption areas. Typically, the fracture pattern does not penetrate below the acrylic block. It is important to note that in the positive control group, where no resorption cavities were present, the supracrestal and subcrestal fracture patterns exhibited similarities. Our study findings corroborate these observations [2]. Although the internal resorption cavities created in the present study were smaller than those used in the model described by Ulusoy et al. [2], the fracture resistance values obtained were nevertheless comparable. One possible explanation is that CSCs may provide a reinforcing effect that reaches a mechanical threshold independent of moderate variations in defect size.

This study is subject to certain limitations, primarily stemming from the use of simulated internal root resorption cavities and the in vitro experimental setting. It is important to recognize that in vitro conditions may not precisely replicate the complexity of clinical scenarios. Furthermore, in the literature, fracture tests have been conducted under various time intervals after force application [21, 30]. Typically, during the early period, no significant differences were observed between the experimental groups and the control group. However, notable differences emerged between these groups with extended time intervals [29]. Therefore, future studies incorporating different waiting times are recommended to gain a more comprehensive understanding of the fracture resistance of CSCs in simulating internal root resorption scenarios. The comparatively lower fracture‐resistance reinforcement observed with NeoMTA Plus and BioMTA+ may be attributed to their lower microhardness, slower or less dense crystalline maturation and more porous final microstructure. These characteristics can reduce their ability to strengthen weakened dentin walls [8, 9].

## 5. Conclusion

Within the limitations of this methodology, it appears that Biodentine may offer greater strength to the tooth structure in terms of fracture resistance when addressing internal root resorption cavities in upper central incisors, surpassing the performance of other CSCs. Importantly, all CSCs demonstrated similar effectiveness in restoring internal root resorption cavities, resulting in comparable fracture resistance. Consequently, BioMTA+ and NeoMTA 2 emerge as potential alternatives for the repair of internal resorption cavities.

## Author Contributions

**Havva Gozde Sen**: conceptualization, methodology, investigation, writing – original draft. **Ayca Yilmaz**: methodology conceptualization, resources, supervision, software.

## Funding

This work was supported by the Scientific Research Projects Coordination Unit of the Istanbul University (Project Number: 37565). The protocol of the work was approved by the Ethics Committee of the University of Istanbul, Turkey (Grant 28678:2020).

## Conflicts of Interest

The authors declare no conflicts of interest.

## Data Availability

The authors have nothing to report.
